# Geographic Differences in Patient Experience After a Diagnosis of Lobular Breast Cancer

**DOI:** 10.1245/s10434-026-19523-2

**Published:** 2026-03-31

**Authors:** Alexis Graham-Stephenson, Claire E. Fanning, Esther L. Geven, Flora Migyanka, Jacqueline S. Jeruss

**Affiliations:** 1 Department of Surgical Oncology, Brigham and Women’s Hospital, Boston, MA USA; 2 University of Michigan Medical School, Ann Arbor, MI USA; 3 Dutch Lobular Breast Cancer Foundation, Sneek, The Netherlands; 4 The Dynami Foundation, Plymouth, MI USA; 5 Department of Surgery and Rogel Cancer Center, University of Michigan, Ann Arbor, MI USA

**Keywords:** Invasive lobular carcinoma, Patient experience, Global health disparities, Psychological stress, Breast cancer, Europe, North America

## Abstract

**Background:**

Invasive lobular breast carcinoma (ILC) has a distinct morphology and complex impact on clinical outcomes. In North America (NA) and Europe, geographic disparities in the management of ILC may contribute to stress experienced by patients. This study examined regional factors associated with ILC diagnosis that affected patient management and survivorship.

**Methods:**

A 31-item questionnaire was distributed to three moderated ILC patient groups. The survey consisted of demographic, Likert, multiple choice, and free response questions. Statistical analysis included chi-squared, Mann–Whitney U, and unpaired two-sample t-testing. P-values <0.05 were significant. Free responses were analyzed for theme identification. The study was exempt from institutional review board approval.

**Results:**

Participants (902) responded from 18 countries in Europe (*n* = 267) and NA (*n* = 635). Stage at diagnosis in Europe vs. NA was I, 23.6 vs. 48.9%; II, 54.1 vs. 30.5%; III, 17.9 vs. 17.0%; and IV, 4.5 vs. 3.7%. European patients had more advanced disease, and initial treatment recommendations more frequently included antihormonal therapy or chemotherapy (*p* <0.001). European patients were less frequently offered lumpectomy (*p* = 0.01). European respondents reported less emotional support from family (*p* = 0.003), employers (*p* = 0.004), and healthcare providers (*p* <0.001). NA respondents reported decreased confidence in the accuracy of medical follow-up (*p* <0.001). Shared themes included anxiety, lack of ILC-specific information relayed by providers, and perceived dismissal by providers.

**Conclusions:**

Although unique differences exist between regions, negative psychological implications associated with ILC are universal. This study provides insight into opportunities for global improvement in ILC management, including improved screening approaches and psychological support.

Invasive lobular breast carcinoma (ILC) is the second most common subtype and accounts for 10–15% of all breast cancer diagnoses.^1,2^ Histologically, the hallmark of ILC is loss of the cell adhesion protein E-cadherin, leading to a single-file pattern of cancer cells.^1–4^ Given this distinct infiltrating growth pattern, ILC remains difficult to detect on exam and mammography (sensitivity of 57–89%).^3,5–7^ As a result, when compared with invasive ductal carcinoma, ILC is more often locally advanced, multifocal, or metastatic at the time of diagnosis.^7–10^ The diagnostic challenges of ILC, inconsistent outcomes data^11^, and relative underrepresentation of patients with ILC in clinical research^12^ creates uncertainty for both patients and providers navigating this diagnosis. This may contribute to the higher mastectomy rates in patients with ILC^2,7–11^, which can have long-term consequences, including patient regret and provider self-reproach.^13^

The uncertainties of an ILC diagnosis also generate significant stress for the team of providers caring for patients with ILC. Breast radiologists have indicated that imaging modalities beyond standard approaches are necessary because of a lack of confidence diagnosing ILC with mammography alone.^7,8^ However, the decreased specificity of magnetic resonance imaging (MRI) can lead to unnecessary biopsies and stress for both patients and radiologists.^7,8,14^ Surgical oncologists also encounter challenges with ILC as a result of the ambiguous tumor size measurement on preoperative imaging, the more frequent positive margin rate than with invasive ductal carcinoma^15,16^, and the difficulty of interpreting intraoperative nodal status. Pathologists can also be tasked with the responsibility of confirming negative margins and lymph node status intraoperatively, which can be challenging given the frequently subtle findings associated with ILC.^13^ Medical oncologists must navigate prognostic discussions with patients in the face of inconsistent outcomes data, limited ILC-specific clinical trial data, and a lack of targeted therapies.^1,11,12,17–19^

The challenges of an ILC diagnosis and management weigh heavily on providers, but it is ultimately patients who endure these impacts. Due to the complexities of an ILC diagnosis^13^, the psychological implications may persist throughout the entire cancer experience.^20^ The ILC patient population is uniquely vulnerable because of heightened concerns over delayed or missed diagnoses^2–5^, the need for additional imaging and procedures, and the inevitable complex surgical decisions that lack a clear optimal treatment approach.^13^ Given the critical and underappreciated challenges of ILC, this study aimed to compare management and patient experiences between Europe and North America (NA) to identify factors that affected the psychological wellbeing of patients with ILC. By focusing on patient perspectives, this study complements work that has emphasized provider perspectives and, in doing so, provides insights to inform patient-centered approaches to ILC care.

## Material and Methods

### Survey Development

A cross-sectional, web-based questionnaire was created to gather information about the experiences of patients diagnosed with ILC. The survey was developed in English by members of the treatment team. The survey was translated to Dutch by a study investigator located in the Netherlands fluent in both English and Dutch. Internal review of the translated version of the survey was performed to ensure clarity of questions and equivalence of concepts. Pilot testing was conducted among study members to confirm usability and functional design.

### Survey Design

The survey consisted of 31 questions using a partially adaptive design, including Likert-scale, multiple-choice, and open-ended response formats. Data collected examined participant demographics, patient experiences with ILC diagnosis and treatment, and psychological aspects of follow-up care.

Before beginning the survey, participants were given an online information sheet explaining the study purpose, procedures, data confidentiality, and voluntary nature of participation. Patients had to agree to participate and give consent to answer the questionnaire. All information was deidentified. The study met criteria for minimal-risk research and was deemed exempt by the University of Michigan institutional review board.

### Participant Recruitment

The survey was hosted on the secure web-based platform SurveyMonkey and distributed to three moderated Facebook patient support groups whose members self-identified as having a diagnosis of ILC. No financial incentives were offered for the study. There was IP restriction (single response) per browser to limit duplicate submissions, and no double entries were included in the dataset.

### Data Analysis

Survey data were analyzed using IBM SPSS Statistics version 26.0 (IBM Corp., Armonk, NY, USA). Descriptive statistics were generated based on the number of survey responses per question. Categorical variables were analyzed using Pearson’s chi-squared test, and Mann–Whitney U testing was used for analysis of non-parametric continuous variables. Two-sided p-values <0.05 were statistically significant.

## Results

### Demographics

A total of 902 respondents from NA (*n* = 635) and the European Union (*n* = 267) completed or partially completed the survey (Table 1). Participants represented 18 countries: the USA, Canada, Austria, Belgium, Denmark, Finland, France, Germany, the Republic of Ireland, Italy, Malta, the Netherlands, Norway, Romania, Serbia, Sweden, Switzerland, and the UK. The median age of respondents was 55 years (interquartile range [IQR] 50–63), and the median age at diagnosis was 52 years (IQR 47–59), with a median of 2 years between diagnosis and survey completion (IQR 1–4). Stage at diagnosis for the overall cohort was as follows: I, 41.6%; II, 37.3%; III: 17.2%; and IV, 3.9%.

**Table 1:** Baseline characteristics of survey respondents (n = 902)

Characteristic	Data
*Geographic region*	
North America	70.4 (635/902)
European Union	29.6 (267/902)
Age, years	55 (50–63)
Age at diagnosis, years	52 (47–59)
Years since diagnosis	2 (1–4)
*Stage at diagnosis*	
I	41.6 (352/847)
II	37.3 (316/847)
III	17.2 (146/847)
IV	3.9 (33/847)

Data are presented as n (%) for categorical variables and median (interquartile range) for continuous variables

### Diagnosis

There were no significant differences in respondent age and age at diagnosis between groups (Table 2). Stage at diagnosis was higher in the European cohort, with 54.1% of respondents presenting with stage II disease; in contrast, 48.9% of NA respondents were initially diagnosed as stage I (p < 0.001). Despite the lower presenting stage among NA respondents, both groups felt that their cancer had been missed on mammogram in the years preceding diagnosis. Rates of stage III and IV disease were similar between groups, regardless of geographic location. Following a new diagnosis, the study groups reported similar rates of active pursual of ILC-specific information. However, NA survey participants were more likely to report receipt of information from the healthcare team related to the specific features and challenges associated with ILC prior to initiating treatment when compared to the European study participants (41.5% vs. 28.2%, *p* < 0.001).

**Table 2:** Survey responses by geographic region

Survey responses	EU	NA	*p*-value
*Demographics*			
Age, years	55 (51–62)	55 (49–64)	0.62
Age at diagnosis, years	51 (47–57)	52 (47–60)	0.13
Years since diagnosis	3 (1–5)	2 (1–4)	**<0.001 (5.147E-4)**
Stage at diagnosis			**<0.001 (2.345E-7)**
I	23.6 (58/246)	48.9 (294/601)	
II	54.1 (133/246)	30.5 (183/601)	
III	17.9 (44/246)	17.0 (102/601)	
IV	4.5 (11/246)	3.7 (22/601)	
*Diagnosis*			
Felt diagnosis was missed on prior mammograms	49.6 (130/262)	53.1 (329/620)	0.35
Informed about the specific features and challenges of ILC by healthcare team prior to treatment	28.2 (74/262)	41.5 (257/620)	**<0.001 (2.1412E-4)**
Actively searched for ILC-specific information after diagnosis	93.5 (245/262)	93.2 (578/620)	0.88
*Treatment*			
*Proposed therapy*			
Lumpectomy	48.7 (127/261)	57.7 (354/614)	**0.01**
Mastectomy	53.3 (139/261)	58.8 (361/614)	0.13
Chemotherapy	48.7 (127/261)	34.5 (212/614)	**<0.001 (8.65E-5)**
Radiation	70.5 (184/261)	64.8 (398/614)	0.10
Anti-hormone therapy	85.1 (222/261)	72.6 (446/614)	**<0.001 (7.66E-5)**
Had breast cancer surgery	91.5 (236/258)	91.3 (553/606)	0.92
Difference between pathology before/after surgery	69.1 (163/236)	68.4 (373/545)	0.86
Increase in cancer stage following surgery	34.3 (58/169)	43.9 (173/394)	**0.03**
Pathology led to change in initial treatment plan	58 (98/169)	49.8 (196/394)	0.07
Required multiple surgeries to obtain clear margins	27.1 (42/155)	17.8 (60/337)	**0.02**
Multiple lumpectomies	9.0 (14/155)	7.4 (25/337)	0.54
Lumpectomy to mastectomy	18.1 (28/155)	10.4 (35/337)	**0.02**
Treatment changes affected level of trust in providers	42.6 (72/169)	31.8 (124/394)	**0.01**
*Follow-up*			
Declared NED	36.9 (94/255)	59.7 (353/591)	**<0.001 (9.7304e-10)**
NED level of confidence (1–5)	3.5 ± 0.96	3.3 ± 0.96	0.09
Confidence in medical follow-up checks (1–5)	3.38 ± 1.01	2.97 ± 1.00	**<0.001 (2.068e-7)**
Local recurrence	4.7 (12/254)	4.8 (28/583)	0.96
Metastatic disease	8.7 (22/254)	9.1 (53/583)	0.72
*Support*			
Sought mental health services to cope with diagnosis	52.0 (132/254)	35.2 (205/583)	**<0.001 (5.1664e-6)**
*How would you rate the emotional support you receive as an ILC patient? (1–5)*			
Family and loved ones	3.69 ± 1.12	3.94 ± 1.06	**0.003**
Friends	3.73 ± 1.00	3.87 ± 0.99	0.073
Work	3.31 ± 1.14	3.57 ± 1.08	**0.004**
Healthcare providers	3.29 ± 1.08	3.71 ± 0.96	**<0.001 (2.535e-6)**

Bold indicates statitically significant *p*-values

Data are presented as n (%) for categorical variables and median (interquartile range) or mean ± standard deviation for continuous variables.

EU, European Union; ILC, invasive lobular breast carcinoma; NA, North America, NED, no evidence of disease.

The study groups reported similar rates of active pursual of ILC-specific information after a new diagnosis. However, NA survey participants were more likely to report receipt of information from the healthcare team related to the specific features and challenges associated with ILC before initiating treatment than were European study participants (41.5% vs. 28.2%, *p* < 0.001).

### Treatment

There was no difference in receipt of surgical treatment between the study groups, with 91.3% of respondents undergoing oncologic breast surgery. Proposed mastectomy rates were not significantly different between geographic region; however, European respondents were less likely to be offered lumpectomy as an initial treatment option (*p* = 0.01). Chemotherapy was proposed more often for European respondents (*p* < 0.001). European patients were also more likely to be offered anti-hormone therapy (*p* < 0.001). Although most patients reported differences in preoperative and postoperative pathology (EU 69.4%, NA 68.4%, *p* = 0.86), NA respondents were more likely to have a resultant increase in cancer stage after surgery (43.9% vs 34.3%, *p* = 0.03). Pathology results led to a change in the initial treatment plan for 52.2% of survey participants. Of the 102 patients (20.7%) requiring additional surgery to obtain negative margins, rates of completion mastectomy were significantly higher among European patients (18.1% vs 10.4%, *p* = 0.02). European participants were also more likely to report that treatment changes affected the level of trust in their healthcare providers (42.6% vs 31.8%, *p* = 0.01).

### Follow-up

After treatment, 447 respondents (52.8%) reported having no evidence of disease (NED), and both study groups conveyed similar levels of confidence in NED status (*p* = 0.09). However, NA respondents reported significantly lower confidence in medical follow-up checks on the Likert scale (score 1–5, average 2.97 vs 3.38, *p* < 0.001). Despite this, there were no significant differences in local recurrence (4.7% vs 4.8%, *p* = 0.96) or metastatic disease (8.7% vs 9.1%, *p* = 0.72) among European and NA survey participants.

### Support

NA respondents reported higher overall levels of support received, including significantly greater support from family and loved ones (*p* = 0.003), work (*p* = 0.004), and healthcare providers (*p* < 0.001). Both groups reported similar degrees of support from friends (*p* = 0.07). In contrast, participants in the European cohort were more likely to seek professional mental health services to cope with an ILC diagnosis (52.0% vs 35.2%, *p* < 0.001).

## Qualitative Analysis

### Negative Experiences

Analysis of free-response questions revealed respondent frustration with routine screening methods, and they described ILC as “sneaky,” “aggressive,” “silent,” “stealthy,” and “elusive” (Table 3). Participants felt that mammograms failed to capture the true extent of disease or missed the finding completely, leading to a false sense of security and, potentially, worse outcomes. As MRI was perceived to offer greater confidence in estimating disease extent at the time of diagnosis, patients reported significant anxiety about the recommendation for standard mammography as the preferred modality for future screening. Survey participants receiving screening MRI exams reported that increased imaging sensitivity resulted in greater peace of mind; others reported continued concern about recurrence or new cancers “hiding,” especially for patients with dense breast tissue. Of patients who underwent systemic staging during workup/treatment, some who received fluorodeoxyglucose-positron emission tomography expressed decreased confidence in the overall accuracy of this imaging modality for the detection of distant ILC.

**Table 3:** Shared themes among survey respondents.

Fear of recurrence	“Because the metastases spread more through the blood and not always through the lymph nodes, it is even more difficult to detect, so it feels like a loaded gun to the back of your head.”^a^ “Because it can come back after years, life is like a kind of fear.”^a^ “It feels like a ticking time bomb.” “It is also unsettling to know that this cancer, unlike ductal, is both difficult to diagnose AND prone to come back even 30 years from original diagnosis. It’s not 5 years and you’re in the clear; the risk of recurrence/metastasis forever looms over you.”
Decreased sensitivity on imaging	“I live in fear everyday knowing that the imaging can’t be picked up.” “What I know now: the image is unreliable and that brings with it a lot of uncertainty.”^a^ “My story is the classic ILC tale of under-diagnosis and doctors’ over dependence on scanning.”
Feelings of isolation associated with an ILC diagnosis	“I feel like I was treated for breast cancer, not ILC.” “Having the less studied/understood form of breast cancer is fundamentally destabilizing and stressful, and this is not properly acknowledged by oncology teams.” “We who have experienced this cancer often feel we are the stepchild in the breast cancer world.”
Limited knowledge resulting in uncertainty	“There is just so little known about ILC that I feel at risk. I fear every day that my treatment regime was not effective and that the ILC will return somewhere else in my body. The lack of knowledge means fear and anxiety.”
Decreased trust in provider team	“I lost confidence in my medical team because they refused to acknowledge the specific features of ILC.” “None of my doctors truly listen.” “I was shocked at how dismissive they were of my cancer.”
Self-advocacy	“As a patient with ILC, I feel strongly that I have to be very assertive with doctors.”^a^ “I had to REALLY advocate for myself to be taken seriously from the get go … it is exhausting.”
Research and information gathering	“Knowledge is power. I felt better informed to make crucial decisions.” “I want to be as well-informed as possible, but I get very scared by what I read, so I just bury my head in the sand again: I find it very difficult to find a good way to deal with this.”
Support groups	“It helped me have a sense of community with other breast cancer patients.” “Support groups with others who have a similar diagnosis was extremely helpful when I was mentally strong enough to seek them out.”

^a^Translated from Dutch.

ILC, invasive lobular breast carcinoma.

Also contributing to patient anxiety was incomplete or absent ILC-specific information relayed by providers. Respondents attributed this communication gap to provider unfamiliarity with the unique features and behavior of lobular cancer, advocating for improved education and research geared toward ILC. The attempt to “downplay” an ILC diagnosis was perceived as provider dismissal, lack of support, or refusal to acknowledge patient concerns about the distinct factors associated with ILC diagnosis and treatment. Although ductal cancer and its associated management were described as “normal,” “generic,” “traditional,” “basic,” and “standard,” respondents sought to distinguish ILC as a separate entity warranting an individual plan for management. For this reason, patients reported seeking multiple opinions, sometimes requiring a change in geographic region, to connect with providers who were knowledgeable about lobular breast cancer and who could adequately address treatment concerns.

### Positive Themes

Despite negative emotions associated with an ILC diagnosis, respondents used their experiences to enact positive change for themselves and the cancer community (Table 3). A shared theme among survey respondents was a strong desire for self-advocacy, with participants requesting additional imaging, genetic testing, surgeries, and novel treatment modalities. Some patients extended this advocacy, founding philanthropic lobular-specific organizations with the goal of funding research and providing patient support. In addition, patients reported seeking support through various organizations and support groups, contributing to a greater sense of community and control and, in turn, feeling better informed to make independent decisions.

## Discussion

This study demonstrated that European patients presented with advanced-stage ILC necessitating chemotherapy more often than patients from NA. Despite these differences, there were universal psychological responses among the European and NA patient groups, suggesting a unique emotional burden to the ILC diagnosis, irrespective of geographic location.

European patients had more advanced disease at the time of diagnosis than did the NA study participants. This discrepancy may be explained by national differences in recommended frequency of screening. In the USA, the National Comprehensive Cancer Network and American Cancer Society recommend annual screening mammography for asymptomatic women aged ≥40 years at average breast cancer risk.^21,22^ Conversely, the European Commission Initiative for Breast Cancer Screening and Diagnosis guidelines recommended biennial mammography screening for asymptomatic women aged 50–69 years with an average risk of breast cancer, and either biennial or triennial mammographic screening for asymptomatic women aged 45–49 with an average risk of breast cancer.^23–25^ The Canadian Task Force on Preventive Health Care recommended either biennial or triennial mammography screening for women aged 50–74 years and optional mammography screening for women aged 40–49 years.^26^ Of the 16 European countries represented in our study, the majority of respondents were from the Netherlands, where national guidelines recommend biennial screening for women beginning at age 50 years.^23,27^ Patients undergoing annual screening mammography have more image-detected tumors, tumors of smaller size, increased surgical options, more effective chemotherapy, and fewer interval cancers than patients undergoing longer screening intervals.^28,29^ Although annual screening in the USA is associated with higher false-negative biopsies and recall rates, this screening approach correspondingly results in more life-years gained and breast cancer-related deaths avoided.^28–32^ The European model for breast cancer screening is through centrally organized population-based programs that focus on equal access and consistent care.^25,30^ In the USA, breast cancer screening is performed as part of a decentralized system that occurs locally after a patient receives an order from a physician, often after a discussion about personal preferences, risk factors, and national guidelines. However, the US insurance-based healthcare system drives disparities in breast cancer screening, with uninsured, underinsured, and low-income individuals facing barriers to care.^33^

The subtle features associated with ILC result in mammographic false-negative rates as high as 43%.^8,10,34^ In a recent survey of 340 breast radiologists, a small fraction of respondents expressed confidence in the ability to identify ILC on screening mammography in the setting of dense breasts, and 79% agreed that supplemental screening was essential given the relatively nuanced and typically noncalcified mammographic appearance of ILC.^7–10,34,35^ MRI has therefore become the preferred approach for both diagnosis and staging of ILC, with sensitivities reported between 93% and 100%.^7–10^ In the USA, MRI is widely incorporated into breast cancer screening protocols and is recommended by both the American Cancer Society and the National Comprehensive Cancer Network annually as an adjunct for high-risk patients.^21,22^ In the USA, geographic access to MRI is generally favorable, with an estimated 70% of US women residing within 20 minutes of an MRI facility.^36^ However, even within the USA, MRI utilization remains somewhat variable depending on geographic location when compared with modalities including ultrasound and mammography.^36^ Comparatively, in Europe, although MRI is increasingly used for screening high-risk women or preoperative staging for specific breast cancer subtypes, availability, reimbursement, and integration into national screening programs differs between countries.^37^ One survey of European breast radiologists found that geographic location influenced the most common indications for breast MRI.^37^ Accordingly, Northern European countries, including the Netherlands, primarily employ MRI for high-risk screening and ILC staging, whereas Southern European countries are more likely to perform preoperative MRI in all patients with cancer.^37^ Moreover, healthcare reimbursement policies vary; in some nations, MRI is fully covered only within organized screening programs, whereas, in other countries, out-of-pocket payment remains common.^38^ Disparities in scanner density across European nations can also contribute to barriers to access, with some countries possessing fewer MRI scanners per capita than others.^38^ Collectively, these regional differences underscore that MRI access and utilization is more variable across Europe than in the USA, potentially delaying ILC detection and contributing to the higher disease stage observed among European respondents in this study. Improved standardization of MRI access across health systems may represent an important step toward mitigating geographic disparities in ILC diagnosis and treatment.

Beyond geographic disparities in screening and diagnosis of breast cancer, there are also significant regional and national differences in ILC management. In this study, European patients were more likely to have chemotherapy and less likely to be offered lumpectomy in their initial treatment recommendations. These findings may reflect the more advanced cancer stage in European patients at the time of diagnosis. Also, antihormonal therapy was more commonly recommended for European patients than for those in NA. This treatment pattern could be indicative of historical differences in breast cancer management between Europe and NA. Relative to the European approach to cancer care, the practice of medical oncology became established at an earlier time point in the USA, with oncologists assuming a primary position on the treatment team with the capacity to prescribe chemotherapy regimens.^39^ In Europe, for a longer period of time, breast cancer treatment was primarily managed by surgeons, who had more experience prescribing less complex antihormonal therapies.^39^

In terms of surgical treatment, there are well-documented differences in mastectomy versus breast conservation surgery rates between European and NA patients. The BrighTNess randomized clinical trial showed that 80% of all eligible European patients opted for lumpectomy compared with 55% of NA patients, irrespective of response to neoadjuvant systemic therapy.^29^ Importantly, in this study, more European patients were recommended to undergo mastectomy, reflecting the distinct aspects of the ILC disease process and this impact on provider recommendations. Simultaneously, within the USA, there have been substantial trends toward higher proportions of lumpectomy-eligible patients undergoing mastectomy.^40,41^ In the US, contralateral prophylactic mastectomy (CPM) rates range from 11% to 56%, despite the lack of evidence-based data to support this procedure outside the setting of a diagnosed high risk for contralateral breast cancer.^40,41^ This CPM trend has not been observed in Europe, where the CPM rate was shown to be stable at 2.6% for the entire patient population, and 7% for patients undergoing ipsilateral mastectomy.^40^ In the USA, patients often have determined their preferred surgical treatment plan before the initial consultation with a breast surgeon, as US patients have access to numerous media sources that promote CPM as a highly preventative, reassuring, and sensible option. In the USA, emphasis is placed on patient autonomy, and the breast surgical oncologist can be limited in redirecting decision-making toward evidence-based care. Conversely, in most European countries, patients more commonly rely on the physician to guide management rather than using alternative resources.^40^

Despite differences in cancer stage and treatment recommendations, patients with ILC across all geographic regions in this study expressed similar psychological themes, including anxiety, misinformation, and perceived dismissal by healthcare providers. Anxiety and heightened psychological distress are extremely prevalent among patients with breast cancer, and levels of patient distress can be highest during transition points in the illness trajectory, such as diagnostic workup, treatment decision-making, follow-up visits, and times of treatment failure.^20,42^ These challenges are amplified by uncertainties that are specific to ILC, including diagnostic delays, lack of targeted therapies, and the lack of ILC-specific knowledge among primary care physicians, who can be a patient’s first point of clinical contact for breast concerns.^1,8,12,13^ Patients with advanced ILC may experience additional anxiety associated with inequitable access to clinical trials. Metastatic breast cancer trial enrollment often requires measurable lesions, and ILC frequently presents with diffuse disease that is not well captured by Response Evaluation Criteria in Solid Tumors.^12^ From a surgical standpoint, there are known higher positive margin rates, necessitating repeat surgeries for patients with ILC because of challenges in characterizing the extent of disease on preoperative imaging, even when utilizing the most sensitive modality, breast MRI.^15,16^ Psychological distress rates for patients with breast cancer undergoing surgery have been reported to be as high as 47% and are twice as high for patients receiving chemotherapy, which can be further magnified by the cognitive side effects of the medication.^20^ Patients with ILC perceive the entire cancer treatment plan to be marked by uncertainty in the face of unclear and sometimes conflicting guidance.^43,44^

When physicians do not effectively communicate the uncertainties regarding the ILC disease process, patients may perceive providers as dismissive, neglectful, uncaring, and untrustworthy. These feelings can be magnified by the factually inaccurate information available on the internet and discussions with uninformed parties in person and online.^45^ Caring for patients with ILC, as described previously, can generate marked angst for surgeons and radiologists, who are expected to achieve precision despite the inherent diagnostic challenges associated with ILC.^13^ Imaging often under- or overestimates tumor extent, leading to unexpected intraoperative findings, positive margins, or the need for additional surgery, which can be demoralizing.^13^ These experiences may erode the surgeon’s sense of professional identity and create a perception of failure, despite the clinician’s best efforts to make an evidence-based treatment plan in the patient’s best interests. Surgeons may then turn to maladaptive coping mechanisms such as emotional withdrawal or overcompensation towards patients, which may represent self-preservation as the result of a perceived failure.^46^ This transference dynamic, in which both the patient and the provider might project their distress onto one another, reflects the complex psychological state associated with caring for and being patients with ILC. This disease presents significant challenges that can threaten the existential identities of those who diagnose, treat, and counsel patients throughout their disease course. Recognizing these vulnerabilities should prompt a broader conversation within the breast oncology community to counsel patients while demonstrating humility, authenticity, and honesty about the formidable challenges of treating ILC.

This study is the first international effort to describe the complex medical, surgical, and psychological experiences of patients with ILC. Further qualitative assessment will be beneficial to help elucidate patient experiences and translate findings into strategies for improved communication, psychological support, and patient-centered care. Although respondents represented 18 countries, the majority were from the USA and the Netherlands, which may limit the generalizability of some findings to other regions. Additionally, the survey was distributed through Facebook patient support groups and therefore captured responses only from individuals with access to and engagement with social media. Responses were also based on patients’ self-report of ILC diagnosis, treatments, and outcomes, which relied on accurate recall and understanding of medical information. Future efforts will focus on the experiences of patients with ILC on a more global scale. Simultaneously, the universal psychological dynamics shared by patients across all countries in this study represent perspectives that are crucial to understanding the ILC experience.

## Conclusions

ILC represents a complex entity that has psychological implications for both patients and providers. In this study, despite geographic differences in disease presentation and management, universal psychological themes emerged, including anxiety, perceived misinformation, loss of bodily autonomy, and distrust in healthcare providers. These findings identified a critical need for both improved ILC-specific diagnostics and therapeutics and also a framework for effective communication that (a) balances honesty with empathy, confidence with humility and (b) acknowledges that a patient’s cancer trajectory is often fraught with uncertainties best managed in the context of patient-centered and medically informed communication.
